# Use of Plasma Therapy for Severe Fever with Thrombocytopenia Syndrome Encephalopathy

**DOI:** 10.3201/eid2207.151791

**Published:** 2016-07

**Authors:** Se Yoon Park, WooYoung Choi, Yong Pil Chong, Sun-Whan Park, Eun Byeol Wang, Won-Ja Lee, Youngmee Jee, Seog-Woon Kwon, Sung-Han Kim

**Affiliations:** Asan Medical Center, University of Ulsan College of Medicine, Seoul, South Korea (S.Y. Park, Y.P. Chong, S.-W. Kwon, S.-H. Kim);; Korea Centers for Disease Control and Prevention, Cheongju, South Korea (W. Choi, S.-W. Park, E.B. Wang, W.-J. Lee, Y. Jee)

**Keywords:** Severe fever with thrombocytopenia syndrome, encephalopathy, plasmapheresis, investigational therapies, viruses, Republic of Korea, South Korea

**To the Editor:** The central nervous system (CNS) manifestations of severe fever with thrombocytopenia syndrome (SFTS) include apathy, seizure, muscular tremor, and coma (*1*,*2*); however, the mechanism underlying CNS manifestations in SFTS is not clear. Deng et al. reported that illness of 15 (13%) of 115 patients with SFTS met the case definition for suspected encephalitis (*1*). However, they did not present any straightforward evidence of CNS invasion by STFS virus (SFTSV). Cui et al. similarly reported that encephalitis developed in one fifth of 538 patients with SFTS (*2*). They found evidence of SFTSV by isolating the virus from the cerebrospinal fluid (CSF) in 1 of 2 patients with SFTS whose CSF was obtained, but they did not mention CSF pleocytosis (*2*). We report a case of SFTS-associated encephalopathy, without pleocytosis and with normal CSF protein and glucose levels, that was confirmed by real-time reverse transcription PCR of the CSF. The patient was treated with experimental plasma exchange followed by convalescent plasma therapy.

During 2015, a 62-year-old woman who had a history of treated tuberculous meningitis 10 years earlier was admitted to a tertiary hospital in Seoul, South Korea (Republic of Korea), with a 5-day fever, myalgia, and headache. On hospital day (HD) 2, CSF examination revealed 1 leukocyte/mm^3^, protein 35 mg/dL (reference 9–58 mg/dL), glucose 74 mg/dL (reference 45–80 mg/dL), and CSF/blood glucose ratio 0.66 (reference 0.50–0.80). No bacteria or fungi were isolated from CSF. On HD 4, her headache worsened, and she displayed confused verbal responses and lacked orientation of time and place. No focal neurologic signs were observed. On HD 5, magnetic resonance imaging of the brain indicated no additional abnormalities of the parenchyma and extra-axial structures except for a focal parenchymal defect in the right midbrain that had been discovered as a sequelae of tuberculous meningitis 10 years earlier. On HD 7, follow-up CSF examination revealed no leukocytes, protein 57 mg/dL, glucose 209 mg/dL, and CSF/blood glucose ratio 0.62. SFTSV was detected by real-time reverse transcription PCR (Technical Appendix) in plasma and CSF (Figure). On HD 8, the patient became comatose and had no eye, verbal, and motor responses to noxious stimuli (Glasgow coma scale 3). Bilateral exotropia was noted with spared light and corneal reflexes and oculocephalic responses. Experimental plasma exchange was performed, and her viral load declined slightly; however, consciousness and platelet count did not change. An ABO-identical nurse who had recovered from SFTS in September 2014 agreed to donate plasma; her indirect immunofluorescence antibody assay (IFA) for SFTSV IgG had been 1:1,024 in October 2014. On HD 17, the patient’s titer of SFTSV IgG was 1:64 before the plasma therapy. We obtained ≈400 mL of convalescent plasma (IFA assay for SFTSV IgG 1:256 at the time of donation) from the donor and transfused it into the patient on HD 17. The viral load in the blood decreased steeply by a factor of 10 (6 × 10^2^ to 6 × 10^1^ copies/mL) during the first 7 hours (4–11 pm on HD 17); it then gradually decreased from 3 × 10^1^ at 7 am on HD 18 to 6 × 10^0^ copies/mL on HD 20, by which time the patient’s mental status had fully recovered (Figure).

**Figure:**
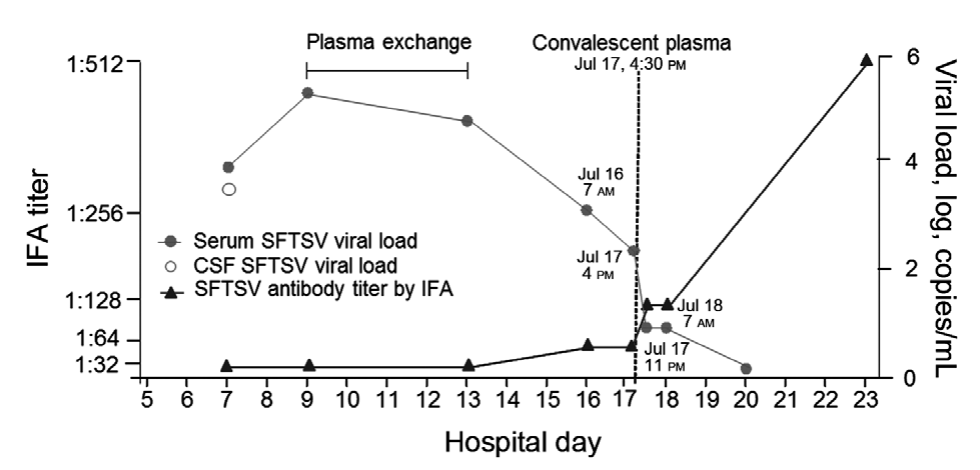
Changes in viral RNA load and immunofluorescence antibody titer and timing of therapies for a 62-year-old woman with SFTSV-associated encephalopathy in response to plasma exchange followed by convalescent plasma therapy, South Korea, 2015. CSF, cerebrospinal fluid; IFA, indirect immunofluorescence antibody assay; SFTSV, severe fever with thrombocytopenia syndrome virus

This case is unique in that SFTS was detected in CSF in the absence of pleocytosis and with normal CSF protein and glucose levels, as in previous reports on influenza-associated acute encephalopathy (*3*). Although headache and encephalitis can occur in patients with SFTS (*1*,*2*), the pathophysiology of CNS manifestations in SFTS is unknown. As with influenza-associated acute encephalopathy, a possible hypothesis is direct invasion of SFTSV into the CNS; another hypothesis is that elevated cytokine levels or renal and hepatic dysfunction are associated with SFTS encephalopathy.

We are aware of 1 report of a favorable outcome of plasma exchange and ribavirin in 2 patients with SFTS and multiorgan failure in South Korea (*4*). However, the patients’ clinical condition did not substantially improve despite the 5-day plasma exchange therapy and viral load only slightly decreased. Use of convalescent plasma therapy in severe acute respiratory syndrome, influenza A(H1N1) and A(H5N1), and Ebola virus disease has been reported (*5*–*7*), but little evidence exists to support its use. However, given the lack of conclusive data, these potential experimental treatments for emerging infectious diseases warrant further study in a clinical trial. Response was favorable in a mouse model of SFTS treated postexposure with antiserum from a patient who had recovered from SFTS (*8*). 

We do not know whether the convalescent plasma therapy given to the patient described here actually had a positive effect because her IFA titer was already increasing around the time she received the plasma therapy. At the time of this writing, 2 patients with SFTS who were treated with intravenous immunoglobulin and corticosteroid had been reported (*9*). Cautious interpretation of these experimental therapies is necessary because these therapies may not have had anything to do with the patients’ recovery.

Technical Appendix Performance of real-time reverse transcription PCR to detect severe fever with thrombocytopenia syndrome virus RNA.
