# Supplementary material for: Use of Plasma Therapy for Severe Fever with Thrombocytopenia Syndrome Encephalopathy
Source: Emerg Infect Dis. 2016 Jul;22(7):1306–8. doi: 10.3201/eid2207.151791 (PMC4918172; doi:10.3201/eid2207.151791)
Supplement: Technical Appendix — Performance of real-time reverse transcription PCR to detect severe fever with thrombocytopenia syndrome virus RNA. [file 15-1791-Techapp-s1.pdf]

Article DOI: http://dx.doi.org/10.3201/eid2207.151791

Use of Plasma Therapy for Severe Fever with Thrombocytopenia Syndrome Encephalopathy

Technical Appendix

Performance of Real-Time Reverse Transcription PCR to Detect Severe Fever with Thrombocytopenia Syndrome Virus RNA

We performed real-time reverse transcription PCR (RT-PCR), as described previously (*1*,*2*), to detect severe fever with thrombocytopenia syndrome virus RNA. RNA was extracted from serum with a viral RNA extraction kit (iNtRON Biotechnology, Gyeonggi, Republic of Korea). The real-time RT-PCR for severe fever with thrombocytopenia syndrome virus was performed in 25 µL reaction mixtures containing 5 µL of RNA template. We used a set of 2 primers (F-GGGTCCCTGAAGGAGTTGTAAA, R-TGCCTTCACCAAGACTATCAATGT) and 1 probe (TexasRed-TTCTGTCTTGCTGGCTCCGCGC-BHQ) for S segment, and a set of 2 primers (F-AAGAAGTGGCTGTTCATCATTATTG, R-GCCTTAAGGACATTGGTGAGTA) and 1 probe (FAM-TCATCCTCCTTGGATATGCAGGCCTCA-BHQ) for M segment. All assays were conducted at the following cycling conditions: 50°C for 30 min (1 cycle); 95°C for 10 min (1 cycle); 95°C for 15 s and 60°C for 45 sec (40 cycles).

References

1. Kim WY, Choi W, Park SW, Wang EB, Lee WJ, Jee Y, et al. Nosocomial transmission of severe fever with thrombocytopenia syndrome in Korea. Clin Infect Dis. 2015;60:1681–3. PubMed http://dx.doi.org/10.1093/cid/civ128

2. Sun Y, Liang M, Qu J, Jin C, Zhang Q, Li J, et al. Early diagnosis of novel SFTS bunyavirus infection by quantitative real-time RT-PCR assay. J Clin Virol. 2012;53:48–53. PubMed http://dx.doi.org/10.1016/j.jcv.2011.09.031
